# Laboratory costs of diagnosing TB in a high multidrug-resistant TB setting

**DOI:** 10.5588/ijtld.20.0586

**Published:** 2021-03-01

**Authors:** L. Cates, V. Crudu, A. Codreanu, N. Ciobanu, H. Fosburgh, T. Cohen, N. A. Menzies

**Affiliations:** 1 Harvard T H Chan School of Public Health, Boston, MA, USA; 2 Chiril Draganiuc Institute of Phthisiopneumology, Chisinau; 3 Nicolae Testemitanu State University of Medicine and Pharmacy, Chisinau, Moldova; 4 Yale University School of Public Health, New Haven, CT, USA

Dear Editor,

Diagnosing drug resistance to TB requires substantial resources, yet there are few unit cost estimates for these services.^1^–^3^ We examined the laboratory costs of diagnosing TB and drug resistance in Moldova, where 29% of treatment-naïve cases have multi-drug-resistant TB (MDR-TB).^4^,^5^ This study was conducted at the Chiril Draganiuc Institute of Phthisiopneumology (IPP) in Chisinau, Moldova, where the National TB Reference Laboratory (NRL) is based. The NRL offers TB microscopy, culture testing and species identification, drug susceptibility testing (DST) and molecular diagnostics.^6^ This allowed cost estimation for a range of tests, including sputum smear microscopy (SSM), Löwenstein-Jenson (LJ) solid culture, BACTEC™ MGIT™ (BD, Franklin Lakes, NJ, USA), Xpert^®^ MTB/RIF (Cepheid, Sunnyvale, CA, USA), phenotypic DST for first- and second-line drugs, and two Hain Lifescience (Nehren, Germany) line-probe assays (LPAs). We assessed costs from a provider perspective from January to December 2018. We included the direct costs of TB diagnostic and monitoring tests, plus laboratory-level overheads. Direct costs included staffing, laboratory equipment, reagents and consumables. Overheads included fuel, repairs and maintenance, office supplies, utilities, buildings, vehicles, recurrent and non-recurrent training courses, quality control, cleaning, management, and general-use laboratory items. We excluded institute-level overheads, research costs and non-laboratory clinical costs, as well as transportation and time costs incurred by patients. For each cost category, we collected data on resource using established methods.^7^ For supplies and equipment, we extracted quantity and price data from accounting and inventory databases. Utilities, buildings, and maintenance costs were extracted from accounting records. Test quantities were extracted from the Moldova National Database for Notification and Follow-Up on Tuberculosis Cases (https://simetb.ifp.md/). Shared costs were directly allocated to different tests and laboratory activity categories, based on interviews with laboratory personnel. Useful life estimates were provided by laboratory personnel (non-recurrent training for 5 years, buildings for 20 years, vehicles for 10 years, laboratory equipment for 2–9 years depending on the item). Costs in Moldovan leu (MDL) were converted to United States dollars (USD) at MDL16.8525=USD1.00 (as of 2 July 2018). We allocated all recurrent and capital costs across the different test types. Overheads were allocated across tests and activities proportional to personnel time spent on each test. The economic cost of capital items was calculated by annuitising the capital cost over the expected useful life, with a 3% discount rate. We analysed these data to describe the distribution of costs across budget categories and test types, the average per-test economic cost (unit cost) for each type of test, and the typical per-patient cost for diagnosis of TB and TB drug resistance. All results are reported in 2018 USD.

The Table shows the distribution of costs across test types, with overhead costs either excluded or included. Based on these analyses, reagents and consumables represented almost half (46.5%) of the total laboratory costs in 2018. This was followed by staff salaries and benefits (19.0%); laboratory equipment (17.3%); buildings (10.3%); fuel, repairs and maintenance (2.5%); and utilities (2.0%). Training, vehicles, quality control and office supplies together accounted for 2.5% of total costs. We calculated unit costs by dividing total test costs, including overheads, by the total tests performed in 2018. These unit costs ranged from less than USD10 per test for LJ and SSM (USD7.59 and USD8.15, respectively) to USD44.78 and USD48.42 for LPA and second-line phenotypic DST panels, respectively. To contextualise our unit cost results, we compared these values to published estimates from a publicly available cost database.^8^ Based on these comparisons, our estimates were similar to published values for SSM, MGIT and Xpert. Our estimates were lower than the range of published values for LJ and first-line phenotypic DST, but higher for LPA. However, there were fewer published values for these tests.^1^,^8^ We found no published estimates for the unit cost of second-line phenotypic DST.

**Table:** Total annual laboratory costs by test type

Test	Cost in USD 2018		Tests or panels conducted in 2018 *n*	Cost/test or /panel (USD 2018)	Average cost of comparative estimates USD (range)

	Excluding overheads USD	Including overheads USD			
SSM	36,971	99,350	12,196	8.15	5.65 (0.20–13.55)
LJ*	76,375	143,432	18,891	7.59	18.80 (12.46–30.42)
MGIT^™^	136,511	231,638	12,044	19.23	19.60 (5.01–39.09)
Xpert^®^ MTB/RIF	221,687	252,876	10,160	30.75	30.69 (4.54–72.56)
First-line DST^†^	86,183	129,848	5,849	22.20	40.47 (26.63–56.97)
Second-line DST^*^	74,480	118,145	2,440^‡^	48.42	NA
LPA^§^	60,428	107,212	2,394	44.78	27.00 (21.54–32.46)
Total cost	692,656	1,082,520			

^*^ Solid media prepared in-house.

^†^ Phenotypic DST costs are reported per-panel. A first-line panel includes isoniazid, rifampicin, ethambutol, streptomycin, and pyrazinamide on liquid culture. A second-line panel includes ethionamide, amikacin, capreomycin, ofloxacin, levofloxacin, moxifloxacin, cycloserine, para-aminosalicyclic acid, kanamycin, linezolid, bedaquilline, and delamanid on liquid culture.

^‡^ 29% of DST panels conducted in 2018 were for second-line DST.

^§^ LPA costs are reported per-panel, and include a first-line panel (GenoType MTBDR*plus* v2.0, Hain Lifescience, Nehren, Germany) and a second-line panel (GenoType MTBDR*sl* v2.0, Hain Lifescience).

USD = US dollar; SSM = sputum smear microscopy; LJ = Löwenstein-Jensen; MGIT = Mycobacteria Growth Indicator Tube; DST = drug susceptibility testing; LPA = line-probe assay.

The per-patient cost of TB diagnosis was calculated by estimating the number of each type of test that would be received by a typical patient (SSM x 1.94, LJ x 1.0, MGIT x 1.0, Xpert x 1.0), multiplied by the unit cost for each test, and summing across all tests. We estimated this cost to be USD73 per patient. Per-patient costs for first- and second-line DST were estimated using a similar approach. Diagnosis of first-line drug resistance involved first-line phenotypic DST, followed by confirmatory LPA for 44% of patients. This gave a cost of USD42 per patient. Diagnosis of second-line drug resistance involved second-line phenotypic DST, followed by confirmatory LPA for 28% of patients. This gave a cost of USD59 per patient. We also estimated the cost per positive diagnosis received by dividing the cost per patient tested by the fraction of patients testing positive. Costs per positive diagnosis were respectively USD913, USD96 and USD221 for patients investigated for TB, first-line DST and second-line DST. The average diagnostic cost per patient suspected of TB, including diagnosis of TB and TB drug resistance, came to USD82.

This study provides empirical estimates for the cost of TB diagnosis in a high MDR-TB setting, including unit costs for individual tests and per-patient costs for common diagnostic scenarios. Given Moldova’s well-developed laboratory infrastructure and distinct testing strategy, care must be taken in interpreting these results for other settings. In particular, the parallel use of SSM, Xpert, LJ and MGIT for initial TB diagnosis is reflected in a high per-patient cost estimate for TB diagnosis. Moreover, studies of health clinics have demonstrated substantial variations between sites, and such site-level variations might also be expected to exist for laboratories.^9^ For this reason, it would not be surprising if other laboratories exhibited different cost structures and unit costs. In addition, we did not consider economies of scale, or conduct sensitivity analyses to understand how costs change based on diagnostic demand. In this setting, testing volume was relatively stable over time, but unit costs might differ with substantial changes in testing volume.

Costs of diagnostic tests are a salient issue among policymakers and TB programme managers.^10^ This study provides unit costs for a wide range of tests collected in a single setting with a consistent costing approach. This allows comparisons of cost between and within different tests, and provides an example of the resources required to sustain a TB laboratory workflow with multiple testing features. These estimates can also assist laboratories in other settings to understand resource needs and to inform cost-effectiveness analyses to identify optimal strategies for detecting and treating TB and drug-resistant TB.
